# LIVER TRANSPLANTATION IN PATIENTS WITH PRIMARY SCLEROSING CHOLANGITIS: A MULTICENTRIC STUDY

**DOI:** 10.1590/0102-672020230051e1769

**Published:** 2023-10-13

**Authors:** Henrique de Aguiar Wiederkehr, Julio Cesar Wiederkehr, Mauro Rafael Da Igreja, Eduardo Brommelstroet Ramos, Marcelo Scheidemantel Nogara, Debora Stroparo Soffiatti, Andrew Massutti, Vivian Laís Sasaki, Barbara de Aguiar Wiederkehr, Igor Raphael Mathias Valejo, Júlio Cezar Uili Coelho

**Affiliations:** 1 Universidade Federal do Paraná, University Hospital, Liver Transplant Department, Curitiba (PR), Brazil;; 2 Santa Isabel Hospital, Liver Transplant Department, Blumenau (SC), Brasil;; 3 Nossa Senhora das Graças Hospital, Liver Transplant Department, Curitiba (PR), Brazil

**Keywords:** Liver transplantation, Cholangitis, sclerosing, Liver cirrhosis, biliary, Transplante de fígado, Colangite esclerosante, Cirrose hepática biliar

## Abstract

**BACKGROUND:**

The prevalence of primary sclerosing cholangitis (PSC) in the general population has not yet been clearly established. The management of PSC should focus on delaying the progression of the disease and restraining its complications. The only curative therapy for the disease remains liver transplantation (LT). PSC is currently the fifth most common indication for LT and corresponds to 5% of all LT indications in adults.

**AIMS:**

Our objective is to evaluate the indications and outcomes of PSC patients undergoing LT in three liver transplantation centers in southern Brazil – Hospital Santa Isabel in Blumenau, Santa Catarina state, and Hospital das Clínicas and Hospital Nossa Senhora das Graças, in Curitiba, Parana state).

**METHODS:**

This is a longitudinal observational study of patients with PSC who underwent LT in three major Brazilian medical centers. Electronic medical records and study protocols of all patients subjected to LT from January 2011 to December 2021 were retrospectively reviewed.

**RESULTS:**

Of the 1,362 transplants performed in the three medical centers, 37 were due to PSC. Recurrence of PSC occurred in three patients (8.1%) in 3.0±2.4 years (range, 1–4 years). The 1-year and 5-year survival rates after the first LT were 83.8 and 80.6%, respectively. The 1-year and 5-year graft survival rates were, respectively, 83.8 and 74.8%.

**CONCLUSIONS:**

Our experience with LT in patients with PSC demonstrated good patient and graft survival results. Most deaths were due to common factors in patients undergoing LT.

## RESUMO

**RACIONAL:**

A prevalência de colangite esclerosante primária (CEP) na população em geral ainda não foi claramente estabelecida. O manejo da CEP deve se concentrar em retardar a progressão da doença e gerenciar suas complicações. A única terapia curativa para a doença continua sendo o transplante hepático (TH). A CEP é atualmente a 5ª indicação mais comum de TH e corresponde a 5% de todas as indicações de TH em adultos

**OBJETIVOS:**

O nosso objetivo é avaliar as indicações e os resultados do transplante de fígado em pacientes com CEP submetidos a TH em três centros de transplante de fígado no sul do Brasil (Hospital Santa Isabel, Blumenau, Santa Catarina; Hospital das Clínicas, Curitiba, Paraná; e Hospital Nossa Senhora das Graças, Curitiba, Paraná).

**MÉTODOS:**

Este é um estudo observacional longitudinal de pacientes com colangite esclerosante primária que foram submetidos a transplante hepático em três grandes centros médicos brasileiros. Os prontuários eletrônicos de todos os pacientes submetidos a TH de Janeiro de 2011 a Dezembro de 2021, foram avaliados retrospectivamente.

**RESULTADOS:**

De um total de 1.362 transplantes realizados nos três centros médicos, 37 eram devidos à CEP. A recorrência de CEP ocorreu em 3 pacientes (8,1%) em 3±2,4 anos (intervalo, 1–4 anos). A taxa de sobrevida de 1 ano e 5 anos após o 1° TH foi respectivamente de 83,8 e 80,6%. As taxas de sobrevida dos enxertos em 1 ano e 5 anos foram, respectivamente, 83,8 e 74,8%.

**CONCLUSÕES:**

A nossa experiência com TH em pacientes com CEP demonstrou bons resultados de sobrevida dos pacientes e dos enxertos. A maioria dos óbitos ocorreu devido a fatores comuns em pacientes submetidos a TH.

## INTRODUCTION

Primary sclerosing cholangitis (PSC) is a chronic progressive cholestatic disease of uncertain etiology characterized by obliterative and concentric fibrosis of the intra and extrahepatic bile ducts, leading to formation of multifocal biliary strictures^12^. Most patients with PSC progress to liver cirrhosis and its severe complications^11^. Although there were reports in the literature about the disease 150 years ago^3^, PSC was only adequately described a few decades ago when endoscopic retrograde cholangiopancreatography (ERCP) and magnetic resonance (MR) cholangiography could completely visualize biliary stenosis. These imaging exams shows bile duct changes that are characterized by short, multifocal, annular strictures that alternate with healthy-looking or slightly dilated ducts. This appearance pattern is called a “bead necklace”. PSC prevalence in the general population has not yet been clearly established. A recent systematic review that included eight studies of North America and Europe estimated that the prevalence rate of the disease is 0.77 per 100,000 people^22^.

The management of PSC should focus on delaying the progression of the disease and restraining its complications, such as obstructive jaundice and liver cirrhosis, which is the final stage of disease progression. The only curative therapy for the disease remains liver transplantation (LT). PSC is currently the fifth most common indication for LT and corresponds to 5% of all LT indications in adults^5^. It is estimated that the overall life expectancy without transplantation is approximately 15 to 20 years after diagnosis^1^. However, survival in PSC patients after LT has increased considerably in the last decade, and presently, the 5-year survival is 85%^3,7^. Biliary stenosis, rejection, and recurrence of PSC are important factors that reduce the quality of life and survival of patients who underwent LT^9^. The recurrence of the disease during the first year after transplantation is low, about 2%; however, about 20 to 37% of patients present recurrence between 3 and 10 years after transplantation^10^. Studies on LT in patients with PSC are scarce in Latin America, with no manuscript published in Brazil^13^.

This study aimed to evaluate the indications and outcomes of LT in patients with PSC who underwent the operation in three major centers in southern Brazil.

## METHODS

The protocol of this study was approved by the Ethics Committee of the University Hospital of the Federal University of Paraná, Brazil (CAAE 59871722.0.1001.0096). This is a longitudinal observational study of patients with PSC who underwent LT in three major Brazilian medical centers — Hospital Santa Isabel, in Blumenau (SC), and Hospital das Clínicas and Hospital Nossa Senhora das Graças, in Curitiba (PR). Electronic medical records and study protocols of all patients subjected to LT from January 2011 to December 2021 were retrospectively reviewed. All patients aged 18 years or older with confirmed diagnosis of PSC were included in the study. Patients with incomplete medical records or missing data were excluded from the sample.

The LT was performed using standard surgical techniques. After the procedure, patients were placed on a standard immunosuppressive protocol consisting of tacrolimus or cyclosporine, azathioprine or mycophenolate mofetil, and prednisone.

Data were obtained regarding patient demographics, diagnosis, Child-Pugh classification per transplant clinical factors, transplant technique, graft function, and complications. For analysis purposes, data were divided into three major groups: preoperative, intraoperative, and postoperative.

Preoperative data included patient demographics Model for End-Stage Liver Disease (MELD) score at the time of transplant and colonoscopy findings to determine the presence of findings compatible with Crohn's disease or ulcerative colitis (UC) disease. Other preoperative data were also analyzed, such as previous diagnosis of Crohn's disease and/or UC and the treatment implemented, date of onset of symptoms of sclerosing cholangitis and date of diagnosis, presence of jaundice, fever, weight loss, and liver cirrhosis and its complications (splenomegaly, hepatomegaly, esophageal varices, portal hypertension, encephalopathy, ascites).

As for the data on preoperative PSC, the following were evaluated: presence of previous percutaneous and endoscopic bile duct treatment and which type was performed; previous surgeries, indication for LT, waiting time, and MELD at the time of entry on the transplant waiting list.

Intraoperative data on LT were analyzed: duration of LT in minutes, duration of cold ischemia time in minutes, duration of warm ischemia time in minutes, type of LT (delivery or cadaveric donor), type performed in the arterial anastomosis, preservation or not of the cava (“piggyback”), and type of biliary anastomosis.

Postoperative data were date of LT, postoperative day at hospital discharge, presence or not of complications including death, immunosuppressive regimen used, recurrence of PSC, presence of ileitis after LT, appearance of colorectal carcinoma, appearance of cholangiocarcinoma (CCA), need for liver re-transplantation and which indication, and outcome.

Recurrence of PSC after LT was defined based on the following criteria:

1. A confirmed diagnosis of PSC before LT,
2. A cholangiogram showing non-anastomotic strictures of the intrahepatic and/or extrahepatic biliary tree with beading and irregularity occurring greater than 90 days post transplantation, or
3. A liver biopsy specimen showing fibrous cholangitis and/or fibro-obliterative lesions with or without ductopenia, biliary fibrosis, or biliary cirrhosis.

Data obtained from the pathological examination of the liver explanted were analyzed, such as the presence of a bile duct tumor in the recipient's liver, a hepatic tumor in the recipient's liver, and changes in the recipient's gallbladder (who was not submitted to a previous cholecystectomy).

### Statistical analysis

Values were expressed as mean ± standard deviation (mean±SD), median, minimum and maximum values, and with a 95% confidence interval (95%CI). Quantitative variables were described as mean, SD, median, minimum, and maximum values. For categorical variables, frequency and percentage were presented.

Kaplan-Meier estimates for the proportion of patients and graft survivors after the first transplant (in years) were presented in tables and graphs. Data were analyzed using the Statistical Package for Social Sciences (SPSS) v.28.0 (Armonk, NY: IBM Corp.). Results with p-value (p)<0.05 (5%) were considered statistically significant.

All data were collected according to the rules previously established by the Ethics Committee, with access to medical records being exclusive to researchers participating in the study and who previously committed to data confidentiality by signing the Term of Commitment to Use of Data (TCUD).

## RESULTS

Of the 1,362 transplants performed in the three medical centers, 37 (2.7%) were due to PSC. The demographic and clinical data of this group are summarized in Table 1.

**Table 1:** Demographic and clinical data of patients with primary sclerosing cholangitis subjected to liver transplantation.

Demographic and clinical data		Number of patients (%)
Gender		
	Male	20 (54)
	Female	17 (46)
Age of transplantation (years)		
	Mean ± standard deviation	40.6±14.9
	Range	18–70
Crohn's disease		2 (5.4)
Ulcerative colitis diagnosis		15 (40.5)
	<1 year before the onset of PSC	4 (26.7)
	>1year after the onset of PSC	11 (73.3)
Treatment of PSC with ursodeoxycholic acid		20 (54.1)
Diagnosis of PSC		
	Liver biopsy and MRCP	22 (59.5%)
	MRCP	14 (37.8%)
	Liver biopsy	1 (2.7%)

PSC: primary sclerosing cholangitis; MRCP: magnetic resonance cholangiopancreatography.

Most of the patients were male (n=20). The mean age at the time of transplantation was 40.6±14.9 years (range, 18–70). In relation to the diagnosis of PSC, 22 patients underwent liver biopsy in addition to magnetic resonance cholangiopancreatography (MRCP). Regarding treatment, 20 patients were using ursodeoxycholic acid, and seven had undergone endoscopic procedures (percutaneous transhepatic cholangiography in three patients and placement of endoscopic stent in four for drainage of the bile duct). Two patients who had endoscopic treatment failure were subjected to surgical bile duct drainage with confection of hepaticojejunostomy in Roux-in-Y.

With respect to associated inflammatory bowel disease (IBD), only two (5.4%) patients had Crohn's disease and 15 (40.5%) had UC. Of those who had UC, the diagnosis preceded the PSC onset in four patients and followed the diagnosis in 11 patients. Of those patients with associated IBD, 13 were receiving only mesalazine as clinical treatment and two were subjected to a left colectomy.

The main symptom of chronic liver disease was jaundice which was presented in 36 (97.3%) patients for 3.0±2.2 years (range, 1–6) pre-transplant. Twelve patients (32.4%) referred weight loss of 10±8 kg, and six (16.2%) had encephalopathy for 1±1 year. A total of 32 (86.5%) patients had non-bleeding esophageal varices and five underwent endoscopic variceal ligation due to acute bleeding. Data of the liver transplants performed are shown in Table 2.

**Table 2:** Data of liver transplantation performed in patients with primary sclerosing cholangitis.

Characteristics		Number of patients (%)
Liver transplant type		
	Deceased donor	36 (97.3)
	Living donor	1 (2.7)
Time between diagnosis of PSC and LT (years)		
	Mean±standart deviation	5.0±6.3
	Range	1–15
MELD score on LT day		
	Mean±standart deviation	23±4.22
	Range	19–36
Indications of LT		
	Chronic liver failure	14 (37.8)
	Recurrent cholangitis	11 (29,7)
	Pruritus	4 (10.8)
	Encephalopathy	4 (10.8)
	Refractory ascites	2 (5.4)
	Portal vein thrombosis	2 (5.4)
Biliary reconstruction		
	Roux-in-Y hepaticojejunostomy	35 (94.6)
	Duct-to-duct	2 (5.4)

PSC: primary sclerosing cholangitis; LT: liver transplantation; MELD: model for end-stage liver disease.

A total of 36 transplants were performed from cadaveric donors (CLT) and one from living-donor (LDLT). The time between the diagnosis of PSC and LT was on average 5.0±6.3 years. The mean MELD score on the LT date was 23±4.22.

The cold ischemia time of the liver was 6.5±3.0 hours and the warm ischemia time was 49±73 minutes. The most common indications for LT were chronic liver failure (37.8%), recurrent cholangitis (29.7%), pruritus (10.8%), and encephalopathy (10.8%) (Table 2).

Biliary reconstruction was performed with hepaticojejunostomy in Roux-en-Y in all patients, except in two with PSC limited to intrahepatic ducts. A duct-to-duct anastomosis was done in these patients.

Data from the postoperative period are shown in Table 3. Early complications were those occurring until the 30^th^ postoperative day or until hospital discharge.

**Table 3:** Postoperative data of patients with primary sclerosing cholangitis subject to liver transplantation.

Postoperative data		Number of patients (%)
Early complications		
	None	19 (51.4)
	Primary graft dysfunction	5 (13.5)
	Bleeding	3 (8.1)
	Biliary anastomosis complications (fistula and stenosis)	4 (10.8)
	Other	6 (16.2%)
Primary sclerosing cholangitis recurrence in 10-year follow-up		3 (8.1)
Time of relapse of primary sclerosing cholangitis after the first transplant (years)*		3.7±2.4 (1–9)
Presence of colorectal neoplasia post-transplant		0 (0)
Presence of cholangiocarcinoma post-transplant		2 (5.4)
Neoplasia in the explant		3 (8.1)
Retransplantation		2 (5.4)
Time between transplant and retransplant (months)*		30.3±26.5; 24.3 (0–73)
Patient survival time after the first transplant (years)*		5,5±4,3; 6 (0–10)
Percentage of patient survival after the first transplant		30 (81)
Graft survival		27 (73.0)
Patient death		10 (27)
Graft survival time after the first transplant (years)*		4.7±3.4; 6 (0–10)

* values presented in years, standard deviation (±) and range.

The most common complications were primary graft dysfunction (n=5) and diffuse bleeding (n=2).

Biliary complications were found in two patients, who were managed conservatively with appropriate drainage and endoscopic papillotomy. Stenosis was treated with endoscopic dilation and biliary stent placement. In one patient with early stenosis, early recurrence of the disease was suspected and confirmed with MRCP. Other complications included cytomegalovirus infection, arterial anastomosis thrombosis, and evisceration^15^.

Recurrence of PSC occurred in three (8.1%) patients in 3±2.4 years (range, 1–4 years). None of the patients had colorectal neoplasia after transplantation.

Re-transplantation was performed in two patients with an interval time between the first transplantation and re-transplantation of 30.3±26.5 months, the indications being rejection of the transplanted organ and recurrence of the disease.

The 1-year and 5-year survival rates after the first LT were 83.8 and 80.6%, respectively, and graft survival rates were, respectively, 83.8 and 74.8%.

In 34 patients, the pathology findings of the explanted liver revealed inflammatory fibrosis of intralobular and septal bile ducts, as well as bile duct obliteration, ductopenia, and biliary cirrhosis: the latter representing the end-stage histological manifestation of PSC.

Localized CCA was found incidentally in three patients (8.1%). All lymph nodes examined were negative for neoplasia. In two patients, CCA was detected in the common bile duct. These two patients developed widespread metastatic disease and died at 38 and 39 months following the LT. The third patient had CCA confined to the gallbladder mucosa and had no recurrence during the follow up of this present study.

### Patient survival after the first transplant

**Figure 1:**
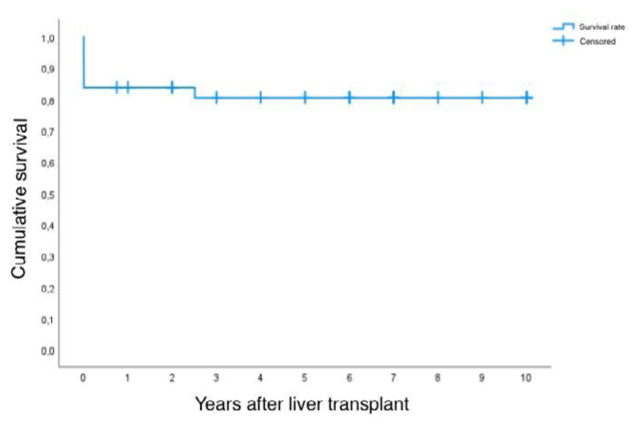
Survival time of post-transplant patients determined by Kaplan-Meier estimate.

Figure 1 shows the Kaplan-Meier estimates for the proportion surviving after the first transplant.

The mean post-transplant survival estimate, considering a follow-up time of 10 years, is 8.1 years, with a CI of 6.9 to 9.4.

### Graft survival rate after the first transplant

**Figure 2:**
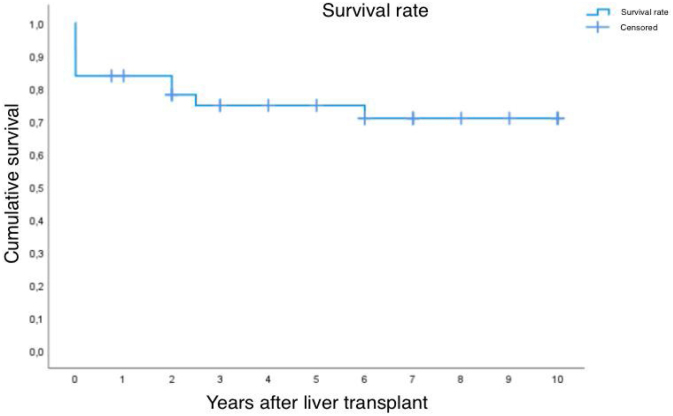
Post-transplant graft survival time determined by Kaplan-Meier estimate.

Figure 2 presents the Kaplan-Meier estimates for the proportion of graft survival after the first transplant, considering as events, the re-transplantation and deaths without re-transplantation.

The mean post-transplant survival estimate, considering a 10-year follow-up, is 7.5 years, with a CI of 6.2 to 8.8 years.

## DISCUSSION

PSC is a rare chronic liver disease in which the inflammatory process and fibrosis of the intra and extrahepatic biliary tree may result in multifocal stenosis of the biliary tree, liver cirrhosis, CCA, and death. Although ursodeoxycholic acid is frequently employed to minimize the PSC clinical manifestations, there is no clinical treatment proven to delay disease progression. LT is the only effective therapy.

In our study, the mean time between the onset of PSC clinical manifestations and LT was 5 years. This time interval was similar to most previous reports from other LT centers^1,5,12^. As in other series, most indications for LT in our patients with PSC were similar to those with other forms of chronic liver disease and relate primarily to impaired quality of life, complications of portal hypertension, and chronic liver failure. Distinctive indications of LT for patients with PSC include recurrent bacterial cholangitis, and intractable pruritus, which were subject to approval by the State Liver Transplantation Committee. In Norway, where there is a high organ donation rate with a waiting list of only one month and a half for LT, PSC patients with cholangiocellular dysplasia confirmed by a biliary brush cytology exam may also receive LT^1^.

The presence of CCA is a formal contraindication for LT by government regulation in Brazil and several other countries because this aggressive cancer is generally associated with very poor post-LT survival. However, in the United States, highly selected patients with unresectable perihilar CCA may undergo LT if they fulfilled a restrictive protocol established by the Mayo Clinic^8^.

CCA is the most common malignancy in PSC^16^. Compared to the general population, patients with PSC have an almost 400-fold increased risk of developing CCA, with the annual incidence estimated at 0.6% per year and a cumulative lifetime risk of 10–0%^12^. Although no patient with known CCA underwent LT in our series, three patients had incidental localized carcinoma of the biliary duct documented on pathologic examination of the explanted liver, two in the common bile duct and one in the gallbladder. And even though all lymph nodes examined were negative, the two patients with common bile duct CCA had widespread recurrence of the tumor and died at 38 and 39 months after LT. The third patient with gallbladder carcinoma was still alive during the 10-year follow-up with no cancer recurrence. Other authors have also reported incidental findings of CCA in the explanted liver, generally with dismal survival due to disseminated recurrence of the carcinoma^19^.

The poor survival of these three patients with incidental and localized CCA was due to the high aggressiveness of the tumor. This finding in our study and in others is important^20^ since it supports the policy of most countries to limit the indication of LT for patients with CCA, even with incidental and localized tumors. These patients shall be subjected to LT only in institutions with very restrictive approved study protocols.

PSC is associated with a high incidence of concomitant IBD, predominantly chronic UC^19^. In our study, 15 (40.5%) patients presented with UC and two (5.4%) with Crohn's disease. Of these patients, five (33.3%) presented with UC after PSC onset. The clinical course of IBD in LT recipients with PSC is highly variable and depends on several factors, including immunosuppressive therapy, incidence of de novo IBD, and diagnosis criteria.

Reported recurrence of PSC after LT varies widely and depends on follow-up time after LT. In a recent meta-analysis with 14 studies on the recurrence of PSC after LT^20^, it was reported a mean recurrence of 17.7% (range, 10.1–27.1%) in a follow-up time range of 2.3–9.2 years. We had a recurrence rate of 8.1% with a mean follow-up time of 10 years (range, 1–9 years) similar to other studies^20^.

Recurrence of PSC has a significant negative impact on both graft and patient survival. In addition, they undergo more re-transplants. In a Japanese nationwide study, Egawa et al.^5^ showed a recurrence rate of 27%, with a graft loss rate of 69% among those with recurring disease. Three patients in our series presented with PSC after LT with a 5-year follow-up. None of these patients underwent re-transplantation due to PSC recurrence.

Considering the technical aspects of LT in patients with PSC, Roux-en-Y hepaticojejunostomy is the preferred biliary anastomosis technique^2,18,21^. However, duct-to-duct anastomosis may also be considered an option when it is feasible^3,14^. Several studies have demonstrated that PSC recurrence after LT is independent of the type of biliary reconstruction if all macroscopic lesions of the biliary duct were completely excised^11^.

Of the 37 patients analyzed, 35 (94.6%) underwent Roux-en-Y hepaticojejunostomy and two (5.4%) underwent duct-to-duct anastomosis. It is worth remarking that in our study, of the 37 transplants we had one living donor transplant.

Our experience with LT for PSC has shown a patient and graft survival rate in 5 years of about 83 and 74.8%, respectively. The European Liver Transplantation Registry demonstrated a 5-year survival rate for patients with PSC of 83%^23^ demonstrating that our data is similar to those of other centers. The present cohort embraces a 20-year period, from 2001 to 2021. During this period, besides the learning curve, several technical improvements were made regarding the procedure, in all centers.

The results of our study regarding patient and graft survival were acceptable and compatible with the literature^4,9,11,18^. The most common cause of death after LT in the present series was primary graft dysfunction and other factors common to patients undergoing LT.

Chronic rejection and recurrence of PSC impact graft survival negatively; however, long-term patient survival can be achieved after re-transplantation. In our study, there were two retransplants whose indication was due to acute or chronic rejection with 100% survival until the end of follow-up. It is worth noting that after a 10-year follow-up, many patients died from other causes unrelated to transplantation, such as cardiovascular diseases, for example. Some studies have suggested that the presence of IBD may increase the risk of rejection^10,24^.

Recurrent disease and chronic rejection were the most common reasons for late graft loss in our series. For the diagnosis of recurrent PSC, as defined by Graziadei et al.^10^, several criteria are considered, such as cholangiography showing intrahepatic and/or extrahepatic biliary structuring, beading and irregularities for at least more than 90 days after LT, and histological findings and confirmed diagnosis of PSC before LT.

The diagnosis of PSC in our series was performed through MRCP demonstrating multifocal constriction mixed with dilated portions of the intra and/or extrahepatic bile duct. The diagnosis could be complemented with liver biopsy demonstrating immunohistochemical ductal proliferation, periductal fibrosis, inflammation, and loss or disappearance of bile ducts. The diagnosis in 22 (59.5%) patients was made with MRCP and liver biopsy, and in 14 (37.8%) patients, only with MRCP.

The patient survival after LT for PSC is favorable, similar to other indications, like alcoholic cirrhosis, viral hepatitis, or hepatocellular carcinoma^9^. Nevertheless, the main factors impairing the long-term outcome after LT for PSC are biliary stricture and recurrent disease^3^. Rates of re-transplantation may be higher owing to recurrent PSC and chronic rejection, yet graft survival remains comparable to other LT indications^17^.

Most of the major clinical indications for LT in the presence of PSC include cirrhosis and its complications such as chronic liver failure, esophageal varices, asthenia, untreatable pruritus, refractory ascites, and encephalopathy, among others^9^. In our study, about 97.3% of the patients had evidence of chronic liver failure and the indications included these complications with recurrent cholangitis (29.7%), which was the most common.

Regarding the allocation of organs for LT in PSC, Brazil's regulatory system uses the same MELD score as other countries^6^. The MELD score above 15 should be used as a cutoff score for an indication with a 5-year survival rate of up to 85%^1^. In our study, we found a mean waiting time for transplantation of 6.6 months and a MELD of 23, similar to other studies in the literature^2^.

## CONCLUSIONS

Our experience with LT in PSC patients demonstrated good patient and graft survival results. Incidental localized CCA found in the explanted liver has an ominous prognosis. Chronic rejection and recurrence of PSC negatively impact graft survival; however, good long-term survival is achievable with re-transplantation.
